# Inhibiting Sphingosine 1-Phosphate Receptor Subtype 3 Attenuates Brain Damage During Ischemia-Reperfusion Injury by Regulating nNOS/NO and Oxidative Stress

**DOI:** 10.3389/fnins.2022.838621

**Published:** 2022-02-15

**Authors:** Xuehui Fan, Hongping Chen, Chen Xu, Yingju Wang, Pengqi Yin, Meng Li, Zhanbin Tang, Fangchao Jiang, Wan Wei, Jihe Song, Guozhong Li, Di Zhong

**Affiliations:** Department of Neurology, The First Affiliated Hospital of Harbin Medical University, Harbin, China

**Keywords:** S1PR3, cerebral ischemia-reperfusion injury, nitric oxide, oxidative stress, CAY-10444

## Abstract

**Background:**

Ischemic stroke (IS) is a common disease endangering human life and health. Cerebral ischemia triggers a series of complex harmful events, including excitotoxicity, inflammation and cell death, as well as increased nitric oxide production through the activation of nitric oxide synthase (NOS). Oxidative stress plays a major role in cerebral ischemia and reperfusion. Sphingosine 1-phosphate receptor subtype 3 (S1PR3), a member of S1P’s G protein-coupled receptors S1PR1-S1PR5, is involved in a variety of biological effects in the body, and its role in regulating oxidative stress during cerebral ischemia and reperfusion is still unclear.

**Methods:**

Transient middle cerebral artery occlusion (tMCAO) mice were selected as the brain ischemia–reperfusion (I/R) injury model. Male C57/BL6 mice were treated with or without a selective S1PR3 inhibition after tMCAO, and changes in infarct volume, Nissl staining, hematoxylin-eosin (H&E) staining and NOS protein, nitric oxide (NO), superoxide dismutase (SOD), and malondialdehyde (MDA) content after tMCAO were observed.

**Results:**

In the cerebral ischemia–reperfusion model, inhibition of S1PR3 improved the infarct volume and neuronal damage in mice after tMCAO. Similarly, inhibition of S1PR3 can reduce the expression of NO synthase subtype neuronal NOS (nNOS) and reduce the production of NO after cerebral ischemia. After cerebral ischemia and reperfusion, the oxidative stress response was enhanced, and after the administration of the S1PR3 inhibitor, the SOD content increased and the MDA content decreased, indicating that S1PR3 plays an important role in regulating oxidative stress response.

**Conclusion:**

Inhibiting S1PR3 attenuates brain damage during I/R injury by regulating nNOS/NO and oxidative stress, which provides a potential new therapeutic target and mechanism for the clinical treatment of IS.

## Introduction

Cerebrovascular disease is the main disease that endangers human health. Ischemic stroke (IS) is the most common type of stroke, accounting for 60–70% of all strokes (Wang et al., 2020). When brain tissue is ischemic for a long period of time, the restoration of blood flow will further damage brain tissue, which is cerebral ischemia–reperfusion (I/R) injury (Taoufik and Probert, 2008). Oxidative stress is a pathophysiological phenomenon in which cells in the body are affected by the outside world, which causes excessive production of reactive oxygen species (ROS), and leads to impairment between oxidation and antioxidant systems, making the system prone to oxidation and cell damage. In cerebral I/R injury, oxidative stress can damage nerve cells through direct damage to reactive oxygen species and activation of other signaling pathways (Frantseva et al., 2001). Nitric oxide (NO) has dual neuroprotective and neurotoxic functions in cerebral ischemic injury (Beray-Berthat et al., 2003), which depends on factors such as the time period after ischemic brain injury, the nitric oxide synthase (NOS) subtype of NO, and the source of cells. Immediately after cerebral ischemia, the NO released by endothelial nitric oxide synthase (eNOS) plays a protective role by promoting vasodilation and inhibiting the aggregation and adhesion of microvessels. However, after the occurrence of cerebral ischemia, NO produced by the excessive activation of neuronal NOS (nNOS) and by later inducible nitric oxide synthase (iNOS) contributes to brain damage (Moro et al., 2004).

Sphingosine 1-phosphate (S1P) is produced by the phosphorylation of sphingosine by sphingosine kinase. S1P is synthesized in the cell and then acts as a bioactive molecule in the extracellular or intracellular pathways. To date, there are five subtypes of S1P receptors: S1P receptor subtype 1 (S1PR1), S1PR2, S1PR3, S1PR4, and S1PR5, among which S1PR1, S1PR2, and S1PR3 are commonly expressed in tissues, S1PR4 is mainly expressed in lymphoid tissues, and S1PR5 is limited to expression in the brain and spleen (Chun et al., 2010). FTY720, a new class of immunomodulator, has an excitatory effect on all four receptor subtypes except S1PR2 (Albert et al., 2005). The study found that S1P and CYM5442 are full agonists for S1PR3 (Wang et al., 2018). FTY720-P is a partial agonist for S1PR3 and requires a certain level of receptor reserve to initiate the response (Stepanovska and Huwiler, 2020). FTY720-P may also inhibit S1P-induced leukocyte rolling and P-selectin mobilization by interfering with S1PR3 (Nussbaum et al., 2015). In addition, TY-52156 and CAY-10444 have been widely used as specific S1PR3 receptor antagonists (Murakami et al., 2010; Li et al., 2015; Shirakawa et al., 2017; Patil et al., 2019). To study the pathophysiological mechanism mediated by S1PR3, these agonists and antagonists have been widely used in experimental studies.

The pathogenicity of S1PR1 in cerebral ischemia is related to neuroinflammation. Inhibition of S1PR1 activity with AUY954 not only alleviated the pro-inflammatory response but also enhanced the anti-inflammatory response after cerebral ischemia. In addition, the regulatory role of S1PR1 in proinflammatory response after cerebral ischemia may be related to the activation of microglia. Such as increasing the number of microglia and cell proliferation, promoting microglia to amoeboid cells transformation (Gaire et al., 2018a,^2019^). Another independent study also suggested that S1PR2 was involved in neuroinflammatory after tMCAO, and S1PR2 may mainly participate in the pro-inflammatory response of activated microglia during cerebral ischemia (Sapkota et al., 2019). However, it is not clear whether S1PR4 or S1PR5 are involved in the pathogenesis of cerebral ischemia. In the mouse brain I/R model, S1PR3 is beneficial to the activation of microglia and polarization of M1-type macrophages (Gaire et al., 2018b). It is still unclear whether S1PR3 is involved in mediating NO production and oxidative stress in I/R. We used the S1PR3 antagonist CAY-10444 to study the role of S1PR3 in cerebral ischemia and reperfusion to provide new methods for the treatment of stroke.

## Materials and Methods

### Animal Studies

For this experiment, C57BL/6 male mice were selected. The mice were SPF grade and weighed approximately 20–25 g. All experimental mice were purchased from Liaoning Changsheng Biotechnology Co., Ltd. All experimental animals were managed and used strictly in accordance with the experimental animal management guidelines of the First Affiliated Hospital of Harbin Medical University, as recommended by the US National Institutes of Health. During the experiment, the mice were housed in an environment with a humidity of 50–60% and a temperature of 23–25°C, the natural circadian rhythm was simulated with a 12/12 h alternating light mode, and the mice were able to eat and drink water freely. Mice are randomly assigned to each group, 4 mice per group.

### Construction of the Transient Middle Cerebral Artery Occlusion Model

Cerebral ischemia was established by generating the tMCAO model using a modified intraluminal technique (Liu et al., 2010). The mice were anesthetized with 3% pentobarbital sodium, their heads were skinned and disinfected, and the anal temperature probe was inserted to keep the body temperature at 37 ± 0.5°C. The skin of the neck was cut open to isolate and expose the common carotid artery, internal carotid artery and external carotid artery. An incision was made on the right common carotid artery, where a 0.21 mm thread was inserted into the internal carotid artery through the common carotid artery until the middle cerebral artery was reached. The depth reached approximately 9 ± 1 mm at the bifurcation of the internal and external carotid arteries. If there was any resistance, the thread was stopped. One hour after ischemia, the thread plug was removed, the skin was sutured, and the mouse was placed on a heating pad. After waking up, the mice were placed in a constant temperature incubator for 24 h. The animals in the sham group were subjected to the same operation process except that the middle cerebral artery (MCA) was not occluded. An inspector unknowingly scored mice for neurological deficits. The deficits were scored as follows: 0, no deficits; 1, forelimb weakness and torso turning to the ipsilateral side when held by tail; 2, unable to extend the opposite forepaw completely; 3, turning to the paralyzed side; 4, dumping to the opposite side; and 5, unable to walk spontaneously, loss of consciousness. It is considered that the tMCAO model is successful when score is 1–4 points. Mice after tMCAO were excluded from this study: (1) one that died before euthanasia; (2) one with a subarachnoid hemorrhage or intraparenchymal hemorrhage; (3) one with a 0 score or 5 score at the time point of euthanasia. When the mice were operated, the neck skin opening is narrowed to reduce the wound surface and unnecessary exposure. The operation is gentle, reducing the physical strain on the tissue. From the beginning of anesthesia to awake, the mice are placed on the 37°C constant temperature heating plate to make the mice in a more comfortable environment, promoting their recovery. Each batch of 20 mice were randomly assigned to 4 cages, and the average success rate of the model was over 90%.CAY-10444 was purchased from Cayman Company and was injected intraperitoneally into mice at 0.5 mg/kg during reperfusion (Gaire et al., 2018b). CAY-10444 was dissolved in dimethylsulfoxide (DMSO, less than 2%). Mice in the V + tMCAO group were intraperitoneally injected with vehicle (DMSO, less than 2%) after ischemia and reperfusion. Mice were randomly divided into the following groups: (1) sham group; (2) 24 h-tMCAO group; (3) CAY-10444 + tMCAO group; and (4) V + tMCAO group.

### 2,3,5-Triphenyltetrazolium Chloride (TTC) Staining

After the mouse was sacrificed, the brain was extracted and cut into seven pieces from the rostral tip (1 mm thick) of the frontal lobe. The tissue was incubated with 2% 2,3,5-triphenyltetrazolium chloride solution (2% TTC, Solarbio) at 37°C in the dark for 30 min and then fixed with 4% paraformaldehyde. Finally, brain slices were imaged with a camera, and the infarct volume was evaluated by ImageJ software. Measure the infarct area and total area of each slice. The infarct volume of each layer is the product of the infarct area and the thickness of the layer. The sum of the infarct volume of each layer is the total infarct volume.

### Nissl Staining, and Hematoxylin-Eosin Staining

The specimens were fixed in 4% buffered formaldehyde, paraffin-embedded and 4 μm thick histological sections were stained with H&E. In Nissl staining, the sections were put into toluidine blue, the staining tank was placed in a constant temperature box at 50–60°C for 25–50 min, 70% ethanol was added for washing, and finally 95% ethanol was used for rapid differentiation. Absolute ethanol dehydrates quickly. The tissue sections were examined and imaged with an optical microscope (Nikon, Y-TV55, JAPAN).

### Immunofluorescent Staining

At the time of 24 h after tMCAO on set, the mice brain tissues were taken and their hearts were perfused. Firstly, pre-cooled saline was used for perfusion and flushing until all blood was released, and then 4% paraformaldehyde was perfused until the mice became stiff. Secondly, the tissues were obtained by dissection at 4°C, fixed in 4% paraformaldehyde for more than 24 h, putting the tissues fixed in paraformaldehyde in a 30% sucrose solution for dehydration for 24–48 h, then it was embedded with OCT and placed in a –80°C refrigerator. The frozen brain tissues were sliced using a cryostat with a thickness of 7 μm, and the slices were directly subjected to immunofluorescence staining. We cover the tissue with 0.5% Triton X-100, permeate it at room temperature for 20 min, and then incubate it with 10% goat serum at room temperature for 1 h (Fulgenzi et al., 2020). The samples were incubated at 4°C overnight with primary antibodies specifically raised against the following proteins: NeuN (Abcam, ab104224, 1:1,000), Iba1 (Abcam, ab178846, 1:500), GFAP (Wanlei, WL0836, 1:100), nNOS (GeneTex, GTX133403, 1:50). Subsequently, the samples were incubated with the appropriate fluorophore-conjugated secondary antibodies (BOSTER, BA1089, 1:100) for 1 h at room temperature in the dark. DAPI (Abcam, ab104139) was used to stain cell nuclei. Images were captured using a fluorescence microscope (Nikon, Y-TV55, JAPAN).

### Fluoro Jade C Staining

Fluoro Jade C (FJC), a polyanionic fluorescein derivative that binds sensitively and selectively to degenerating neurons, was used to examine dynamic time-course changes in dying neurons in the brains of the animal models described above (Schmued et al., 2005). Sections were first treated as for IF staining, and then FJC staining was performed. We immersed the slides in 80% ethanol solution containing 1% NaOH for 5 min. They were rinsed in 70% ethanol for 2 min, then incubated them in 0.06% potassium permanganate solution for 10 min. After rinsing with distilled water for 2 min, the treated slides were stained in 0.0001% concentration of FJ-C (United States, Biosensis) solution for 10 min, adding Solution D (DAPI) to the above FJC solution. Finally, Slides were mounted with DPX, sections were examined under a fluorescence microscope and images were captured for demonstration.

### Western Blot Analysis

Brain tissue from the right hemisphere was obtained, and proteins were extracted on the first day after I/R. The protein concentration of the samples was determined by the BCA protein detection Kit. In addition, 30 μg of protein from each group were loaded onto an 7.5 or 10% SDS–PAGE gel. After electrophoresis, the brain proteins were transferred to polyvinylidene fluoride (PVDF) membranes, blocked with 5% skim milk at room temperature for 1 h, and then incubated with the primary antibody overnight in a 4°C refrigerator. After an incubation with goat anti-mouse and anti-rabbit (Abmart, M21003, 1:2,000) secondary antibodies for 45 min at room temperature, membranes were washed with TBST, and then incubated with enhanced chemiluminescence (ECL) reagent (biosharp, BL520A, China) for detection. Primary antibodies included: anti-iNOS (1:1,000, 22226-1-AP, Proteintech, United States), anti-nNOS (1:1,000, ab76067, Abcam, United States, United States), and anti-eNOS (1:1,000, ab199956, Abcam, United States). β-Tubulin (1:1,000, 10094-1-AP, Proteintech, United States) was used for internal comparison. ImageJ software was used to quantitatively analyze the gray values of all protein bands.

### Nitric Oxide Detection

NO detection kit was purchased from Nanjing Jiancheng Institute of Bioengineering. Since NO metabolism will eventually lead to the production of nitrite, the NO content is measured by quantifying the levels of nitrate and nitrite in the sample. To this end, cadmium is used to convert nitrate to nitrite, then the Griess reaction is performed, and the NO content in each sample is measured using a microplate reader at 570 nm.

### Superoxide Dismutase and Malondialdehyde Detection

A commercial kit (Wanleibio, Shenyang, China) was used to measure MDA and SOD levels in the right brain tissue of mice. All measurements were performed in accordance with the manufacturer’s instructions. MDA and SOD were determined by the absorbance at 532 and 570 nm, respectively.

### Statistical Analysis

Use GraphPad Prism 8.0 statistical software for statistical analysis, and the experimental data are expressed as mean ± SEM. Differences between groups were analyzed using one-way ANOVA followed by the Tukey *post hoc* test. *P* < 0.05 was regarded as statistically significant.

## Results

### Inhibition of Sphingosine 1-Phosphate Receptor Subtype 3 Can Improve Infarct Volume and Neuron Damage in Mice After Transient Middle Cerebral Artery Occlusion

**FIGURE 1:**
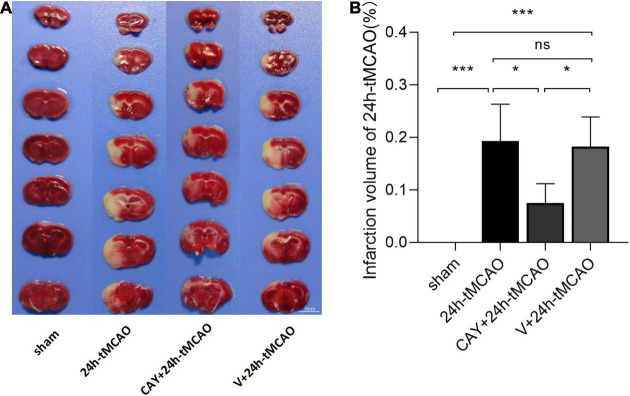
Infarction volume of mice subjected to tMCAO after administration of an S1PR3-specific antagonist. **(A,B)** Representative images and statistical results of TTC staining of brain tissues in different groups (*n* = 4). Scale bar = 5 mm. Data are presented as the mean ± SEM. *P*-values were determined by ANOVA followed by the Tukey *post hoc* test, *P < 0.05; ***P < 0.001; and ns, not significant.

**FIGURE 2:**
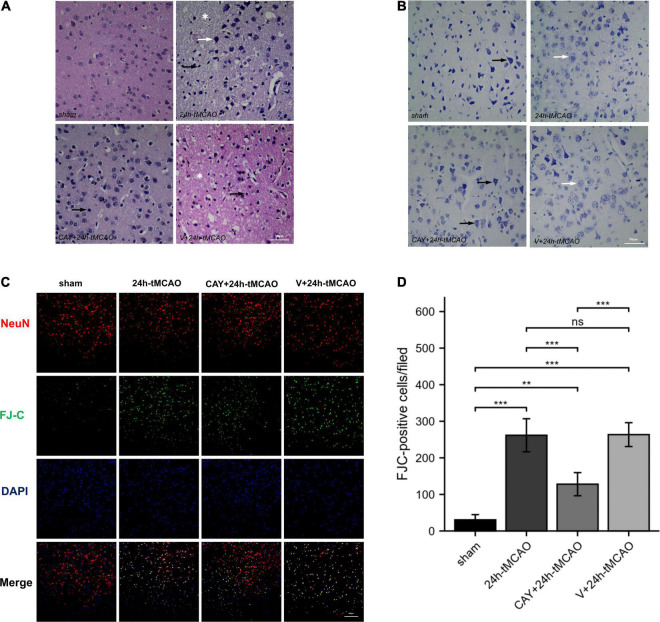
H&E, Nissl and FJC staining in brain tissue of mice subjected to tMCAO after administration of an S1PR3-specific antagonist. **(A)** H&E staining in brain tissue of mice subjected to tMCAO after administration of an S1PR3-specific antagonist. Vacuolated neuropil in brain tissue (*). The black arrow points to the glial cells and the white arrow points to the nucleus of neurons that were reduced and trachychromatic (→), *n* = 4. Scale bar = 50 μm. **(B)** Nissl staining in brain tissue of mice subjected to tMCAO after administration of an S1PR3-specific antagonist. The black arrow points to neurons with Nissl bodies. The white arrow points to neurons with absent Nissl bodies, *n* = 4. Scale bar = 50 μm. **(C,D)** DAPI (blue)/FJC (green)/NeuN (red) Representative immunofluorescence images and statistical results of mouse brain tissue slices after tMCAO (*n* = 4). Data are presented as the mean ± SEM. *P*-values were determined by ANOVA followed by the Tukey *post hoc* test, **P < 0.01; ***P < 0.001; and ns, not significant. Scale bar = 100 μm.

**FIGURE 3:**
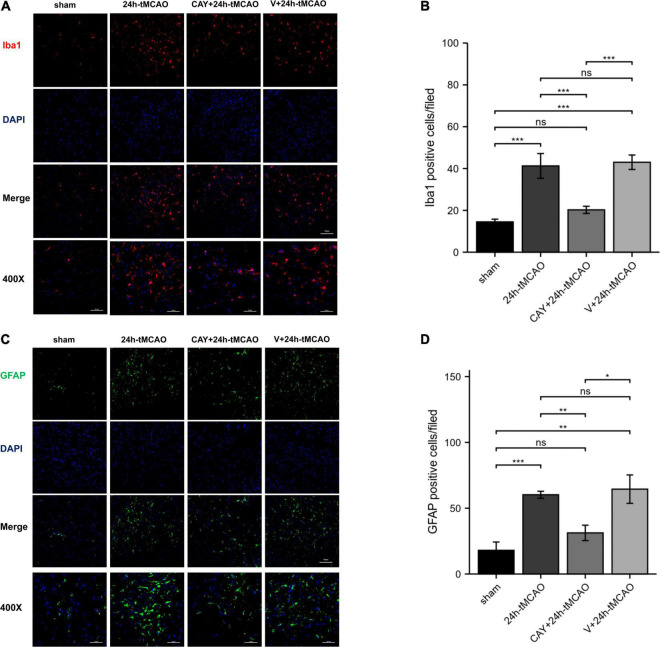
Inhibition of S1PR3 attenuates microglia and astrocyte activation after tMCAO. **(A,B)** DAPI (blue)/Iba1 (red) Representative immunofluorescence images and statistical results of mouse brain tissue slices after tMCAO (*n* = 4). **(C,D)** DAPI (blue)/GFAP (green) Representative immunofluorescence images and statistical results of mouse brain tissue slices after tMCAO (*n* = 4). Data are presented as the mean ± SEM. *P*-values were determined by ANOVA followed by the Tukey *post hoc* test, *P < 0.05; **P < 0.01; ***P < 0.001; and ns, not significant. Scale bar = 100 μm. 400×, Scale bar = 50 μm.

In our previous studies, we found that the expression of S1PR3 was highest 24 h after tMCAO and then decreased, so we chose to extract the brain 24 h after tMCAO. To confirm that inhibition of S1PR3 can reduce cerebral I/R injury, we performed TTC staining. Compared with the 24 h tMCAO group, the cerebral infarction volume of mice was reduced following CAY-10444 administration (*P* < 0.05) (Figures 1A,B). The brain tissue morphology of mice was examined after tMCAO. H&E staining showed that the tissue surrounding the infarct was damaged after tMCAO, the peripheral neuron was characterized by nuclear pyknosis, the staining was darker, the penumbra area was swollen, neuropil vacuolation, glial cell hyperplasia. In the pyramidal cell layer and granular layer of the cerebral cortex, H&E staining showed that CAY + 24 h-tMCAO mice had pyknosis and deep staining of nuclei around the infarct, the number of unclear structures decreased, the neuropil vacuolation of the infarct focus are alleviated, and the number of glial cells around the infarct was also reduced (Figure 2A). Nissl staining of mice after tMCAO showed the disappearance of Nissl bodies in neurons. Compared with the 24 h tMCAO group, there were more Nissl bodies in neurons in the CAY + 24 h tMCAO group (Figure 2B). Fluoro-Jade C staining was performed on mice brain tissues. The results showed that the number of Fluoro-Jade C-positive cells in the 24 h-tMCAO group and the V + 24 h-tMCAO group increased significantly (*P* < 0.001 and *P* < 0.001, respectively), while the number of Fluoro-Jade C-positive cells of the mice treated with CAY-10444 significantly decreased (*P* < 0.001 and *P* < 0.001, respectively) (Figures 2C,D). Subsequently, we stained brain tissues for Iba1 and GFAP, we found that the number of Iba1-positive cells around the infarct area increased significantly after tMCAO (*p* < 0.001), showing amoeboid-like changes. After administrating of CAY-10444, the number of Iba1-positive cells decreased (*p* < 0.001) (Figures 3A,B), the number of amoeboid microglia also significantly reduced. Compared with the Sham group, the number of GFAP-positive cells increased significantly after tMCAO in mice (*p* < 0.001), while the number of GFAP-positive cells showed a decreasing trend after CAY-10444 was administered (*p* < 0.01) (Figures 3C,D).

### Inhibition of Sphingosine 1-Phosphate Receptor Subtype 3 Can Inhibit the Expression of Neuronal NOS After Ischemia–Reperfusion

**FIGURE 4:**
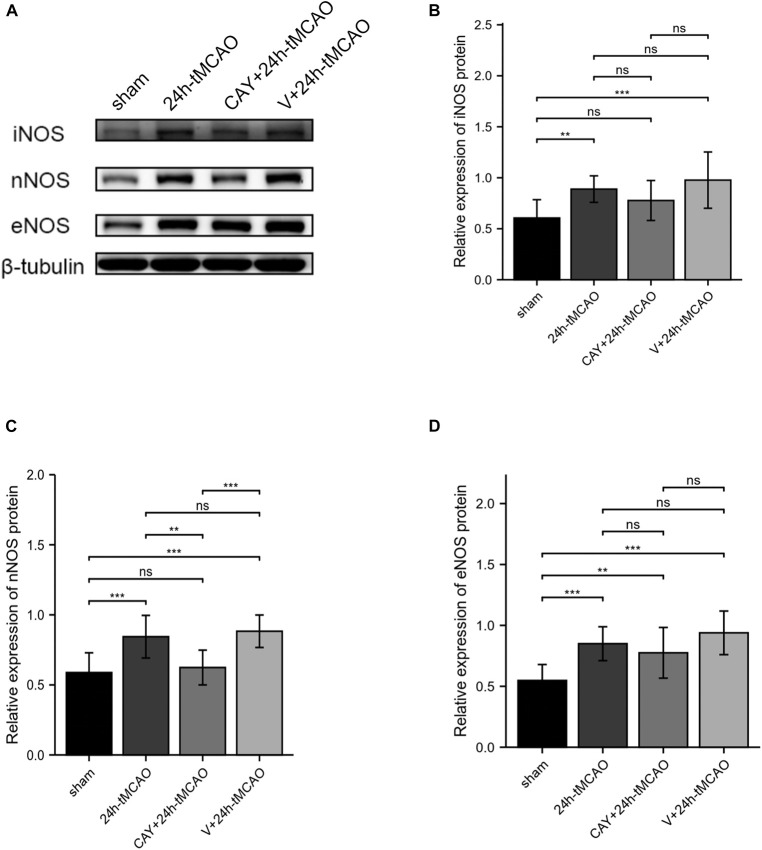
Inhibition of S1PR3 inhibited the expression of nNOS after I/R. **(A)** Western blot of the sham group, 24 h tMCAO group, CAY10444 + 24 h tMCAO group, V + 24 h tMCAO group, and iNOS, nNOS and eNOS expression. **(B–D)** The expression levels of iNOS, nNOS and eNOS proteins (*n* = 4). Data are presented as the mean ± SEM. *P*-values were determined by ANOVA followed by the Tukey *post hoc* test, **P < 0.01; ***P < 0.001; and ns, not significant.

**FIGURE 5:**
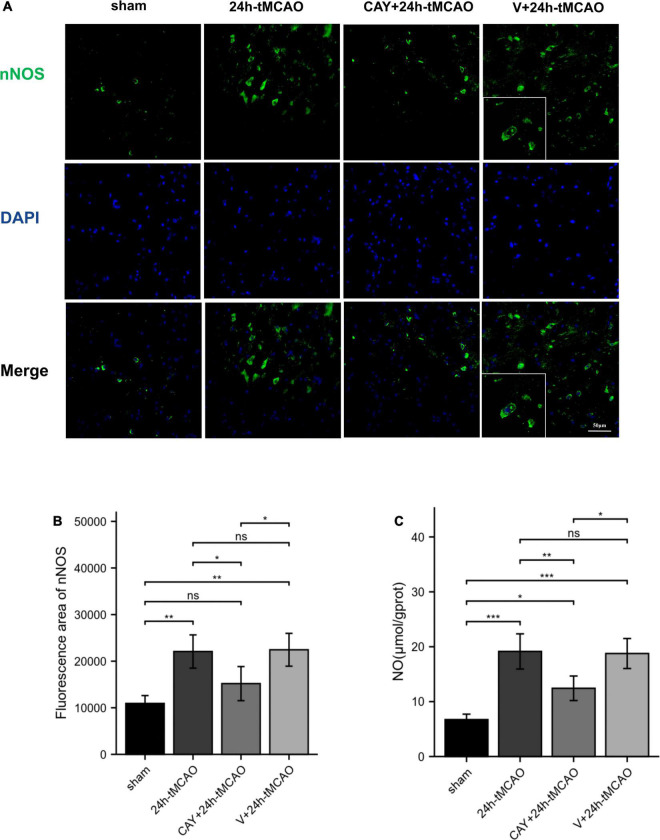
nNOS fluorescence image and NO content expression. **(A,B)** DAPI (blue)/nNOS (green) Representative immunofluorescence images and statistical results of mouse brain tissue slices after tMCAO. **(C)** NO production was measured (*n* = 4). Data are presented as the mean ± SEM. *P*-values were determined by ANOVA followed by the Tukey *post hoc* test, *P < 0.05; **P < 0.01; ***P < 0.001; and ns, not significant.

To confirm the effect of S1PR3 on the expression of nNOS, iNOS and eNOS proteins after cerebral I/R, we used Western blotting to detect the expression of related proteins 24 h after I/R. As shown in Figure 4, the expression of nNOS protein in the tMCAO group increased (*p* < 0.001), and after CAY-10444 was administered, the expression decreased (*p* < 0.01) (Figure 4C). After tMCAO, the expression of iNOS and eNOS both increased (*p* < 0.01 and *p* < 0.001, respectively) (Figures 4B,D). After CAY-10444 administration, the expression of iNOS and eNOS did not change significantly (*P* > 0.05) (Figures 4B,D). Subsequently, we performed immunofluorescence staining on mouse brain slices (Figure 5A). The results showed that 24 h after tMCAO, there was more fluorescent staining of nNOS in the peri-ischemic regions (*p* < 0.01). After CAY-10444 administration, nNOS fluorescence staining was reduced (*p* < 0.05) (Figure 5B). This shows that after tMCAO, inhibition of S1PR3 reduces the expression of nNOS.

### Inhibition of Sphingosine 1-Phosphate Receptor Subtype 3 Can Inhibit the Formation of Nitric Oxide After Ischemia–Reperfusion

To determine whether S1PR3 mediates the production of NO and causes brain damage, we measured the content of nitric oxide in brain tissue. NO content determination in brain tissue showed that after 24 h of I/R, the NO level in the tMCAO group was significantly higher than that in the sham group (*p* < 0.001). Compared with 24 h-tMCAO and V + 24 h-tMCAO, the NO level of the CAY-10444 + 24 h-tMCAO group was significantly lower (*p* < 0.01) (Figure 5C). This suggests that in I/R injury, S1PR3 regulates the production of NO by regulating nNOS.

### Sphingosine 1-Phosphate Receptor Subtype 3 Participates in the Regulation of Superoxide Dismutase and Malondialdehyde After Ischemia–Reperfusion

**FIGURE 6:**
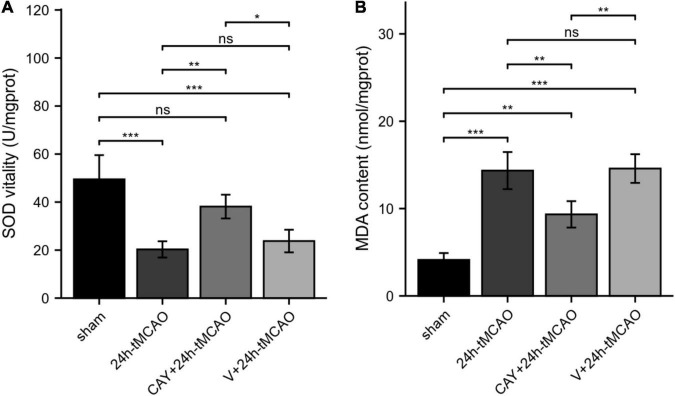
SOD vitality and MDA content in mice subjected to tMCAO. **(A)** SOD vitality was measured (*n* = 4). **(B)** MDA content was measured (*n* = 4). Data are presented as the mean ± SEM. *P*-values were determined by ANOVA followed by the Tukey *post hoc* test, *P < 0.05; **P < 0.01; ***P < 0.001; and ns, not significant.

To study whether S1PR3 is involved in the oxidative stress response in the brain tissue of tMCAO model mice, we examined the changes in SOD vitality and MDA content in brain tissue after brain I/R. Compared with the sham group, the SOD vitality in the brain tissue of mice in the 24 h-tMCAO group was significantly reduced (*p* < 0.001). Compared with that of the 24 h tMCAO group, the SOD vitality of the CAY + 24 h tMCAO group was significantly higher (*p* < 0.01) (Figure 6A). MDA content determination results showed that compared with the sham group, the MDA content in the brain tissue of the 24 h-tMCAO group increased significantly up (*p* < 0.001) and compared with the 24 h-tMCAO group, the MDA content of the CAY + 24 h-tMCAO group was significantly decreased (*p* < 0.01) (Figure 6B). These results show that S1PR3 is involved in the regulation of oxidative stress after I/R.

## Discussion

Studies have found that S1PR3 plays a role in cell inflammation, cell proliferation, cell migration, tumor invasion, I/R, tissue fibrosis, and vascular activity (Fan et al., 2021). In an *in vivo* mouse model of myocardial I/R, it was observed that high-density lipoprotein and its component S1P protect the heart from I/R damage through an independent signaling pathway mediated by S1PR3 (Levkau et al., 2004). S1PR3(–/–) mice are protected from kidney I/R damage through mechanisms involving bone marrow-derived dendritic cells (BMDCs) and their immunomodulatory functions (Bajwa et al., 2012). Bajwa et al. (2016) found that adoptively transferred S1PR3(–/–) BMDCs prevent kidney I/R damage through interaction in the spleen and expansion of splenic CD4 + Foxp3 + T regulatory cells (Tregs). In contrast to the protective effect on heart I/R, S1PR3 shows the opposite effect on kidney I/R, which is speculated to be due to the existence of cells and tissues at different developmental stages of disease. S1PR1 and S1PR2 has previously been found to be involved in the activation of microglia during cerebral ischemia reperfusion (Gaire et al., 2018a,^2019^; Sapkota et al., 2019). A recent study showed that S1PR3 contributes to the activation of microglia and the polarization of M1 macrophages in a mouse brain I/R model (Gaire et al., 2018b). In our study, we found that inhibition of S1PR3 reduced I/R injury, which was confirmed by the reduction of infarct volume. The results of H&E, Nissl and FJC staining confirmed the point that S1PR3 mediates brain damage during cerebral ischemia and reperfusion.

nNOS mediates early neurological damage, and overexpression of nNOS plays a key role in the early stages of ischemia and excitotoxic injury (Von Arnim et al., 2001). iNOS subsequently increased, and both changes have adverse effects on cerebral ischemia. The production of nitric oxide (NO) is one branch of the ornithine cycle, which is catalyzed by L-arginine and oxygen NOS. NO can react with superoxide to form peroxynitrite (ONOO), which is an effective and destructive oxidant (Zhang et al., 2018). During ischemia, the NO produced by nNOS and iNOS may be neurotoxic, partly because the formation of peroxynitrite free radicals causes direct damage to mitochondrial enzymes and DNA (Zhao et al., 2000; Sims and Anderson, 2002). In addition, the increase in NO produced by nNOS or iNOS can promote ischemic damage through free radical damage, tissue inflammation and microcirculation failure (Iadecola, 1997). In a cerebral ischemia model, nNOS knockout mice showed smaller infarct sizes and fewer neurological defects after middle cerebral artery occlusion (Nakamura et al., 2015). Compared with wild-type mice, mice lacking the iNOS gene showed fewer neurological deficits and infarct volumes after MCAO (Yang et al., 2019). In our experiment, the expression of nNOS and iNOS was higher than that of the sham group after cerebral ischemia, which is consistent with previous studies. After inhibiting S1PR3, we found reduced nNOS expression, and significantly reduced NO content. Heo and Im (2019) found that inhibiting S1PR3 can reduce the expression of LPS-induced inflammatory genes, such as iNOS and cyclooxygenase-2 (COX-2). However, our research found that inhibition of S1PR3 did not reduce the expression of iNOS, indicating that S1PR3 reduces the expression of NO by reducing nNOS.

Studies have shown that eNOS protein expression in cerebral blood vessels after focal cerebral ischemia protects against cerebral ischemia by protecting cerebral blood flow (Muid et al., 2016). Lv et al. (2020) found that sphingosine kinase 1 (Sphk1)/S1P signaling may mediate angiogenesis after cerebral ischemia by regulating eNOS activity and NO production. We found that the expression of eNOS increased after cerebral ischemia and reperfusion, but inhibition of S1PR3 did not affect the expression of eNOS, suggesting that S1PR3 does not play a relevant role in regulating eNOS activity in the tMCAO model.

We previously mentioned that S1PR3 is involved in regulating the production of NO and that NO is involved in excitotoxicity. During cerebral ischemia, NO can mediate glutamate neurotoxicity in cortical and hippocampal neurons. After cerebral ischemic attack, oxidative stress plays a major role in neuroinflammatory diseases. Mitochondria play a key role in energy metabolism in the body. When energy metabolism is dysregulated, mitochondria produce a large amount of ROS and cause tissue oxidative stress damage (Hussain et al., 2018). Under normal physiological conditions, SOD (superoxide dismutase), GPX (glutathione peroxidase), catalase and other antioxidant enzymes can protect brain tissue from ROS poisoning through catalysis and maintain sexual balance (Ouyang et al., 2015; Zhang et al., 2016). During cerebral ischemia and reperfusion, the production of ROS is significantly increased, and SOD can be consumed by catalase reactions. As a result, the body’s oxidation and anti-oxidation balance is broken, making the body more susceptible to oxidation and causing cells to undergo oxidative damage (Wang et al., 2019). MDA (malondialdehyde) is an indicator for the severity of oxidative stress within the tissue, and the level of MDA can indirectly measure the degree of tissue damage. High blood lipid levels and high oxygen consumption are the causes of brain oxidative stress damage (Ozkul et al., 2007). In our study, after cerebral ischemia, the activity of SOD decreased, and the content of MDA increased. After the S1PR3 inhibitor CAY-10444 was administered to tMCAO mice, the MDA content in the brain tissues decreased significantly, and the SOD vitality increased, indicating that S1PR3 is involved in the regulation of cerebral ischemia and oxidative stress. Previous studies have found that S1P induces NADPH oxidase activity and intracellular ROS generation in a time-dependent manner (Lin et al., 2016). Therefore, more research is needed to confirm whether S1P is an oxidative stress process regulated by S1PR3 during cerebral ischemia and reperfusion.

CAY10444 has been widely used as a specific antagonist of S1PR3, but other modes of action have been found. Previous studies have found that CAY10444 (10 μM) inhibits [Ca^2+^]i increases via purinergic P2 receptor or α1A-adrenoceptor stimulation and α1A-adrenoceptor-mediated contraction, while not affecting the S1PR3-mediated decrease of forskolin-induced cAMP accumulation (Jongsma et al., 2006). The proliferation of ovarian cancer cells was not affected by S1PR3 inhibitor CAY10444 (1 μM) (Illuzzi et al., 2010). S1PR3 specific inhibitor CAY10444 (10 μM) showed no effect on the protection of platelet-activating factor induced mesenteric venular microvascular permeability by S1P (Zhang et al., 2010). The concentrations of CAY-10444 used in these studies may be too low, mostly 1 or 10 μm, to significantly block the S1PR3 receptor. In previous studies, the use of CAY10444 reduced the polarization of microglia and proved the effectiveness of the inhibitor for this model 30305119 (Gaire et al., 2018b). Therefore, we chose this concentration for the experiment. However, it is necessary to use gene knockout in future research.

## Conclusion

**FIGURE 7:**
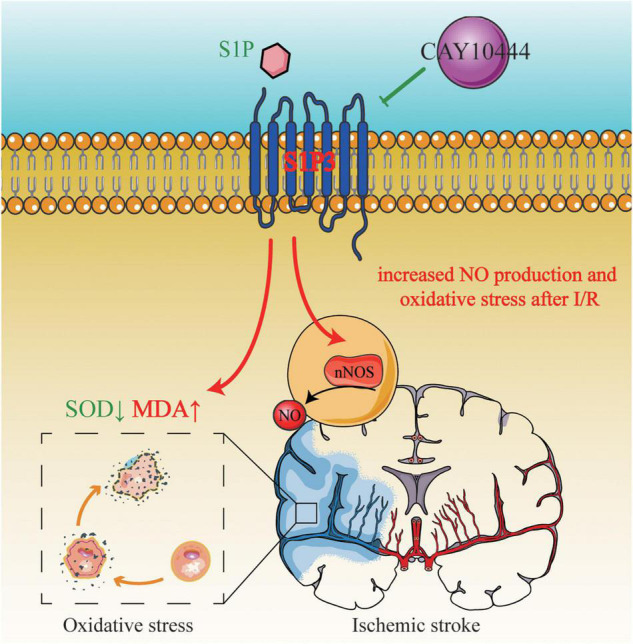
S1PR3 mediates nNOS/NO and oxidative stress during cerebral ischemia and reperfusion.

In summary, our results provide a new evidence that S1PR3 participates in the regulation of oxidative stress after cerebral ischemia and reperfusion and regulates brain damage after cerebral ischemia through regulation of nNOS/NO. Figure 7 shows that S1PR3 mediates nNOS/NO and oxidative stress during cerebral ischemia-reperfusion. The results of this study provide new potential therapeutic targets and mechanisms for the treatment of cerebral ischemia and reperfusion injury. These results may provide pharmacological evidence for the potential application of CAY-10444 in the treatment of cerebral ischemic injury and as an effective treatment for IS.

## Data Availability Statement

The original contributions presented in the study are included in the article/supplementary material, further inquiries can be directed to the corresponding author/s.

## Ethics Statement

The animal study was reviewed and approved by the Laboratory Animals Ethics Committee of the First Affiliated Hospital of Harbin Medical University.

## Author Contributions

XF performed experiments and wrote the manuscript. HC conceived the idea of the study. CX, YW, PY, and ML designed the experiments. ZT, FJ, WW, and JS analyzed the data. GL and DZ revised the final manuscript. All authors contributed to the article and approved the submitted version.

## Conflict of Interest

The authors declare that the research was conducted in the absence of any commercial or financial relationships that could be construed as a potential conflict of interest.

## Publisher’s Note

All claims expressed in this article are solely those of the authors and do not necessarily represent those of their affiliated organizations, or those of the publisher, the editors and the reviewers. Any product that may be evaluated in this article, or claim that may be made by its manufacturer, is not guaranteed or endorsed by the publisher.
